# Physical activity and the risk of developing 8 age-related diseases: epidemiological and Mendelian randomization studies

**DOI:** 10.1186/s11556-024-00359-2

**Published:** 2024-09-18

**Authors:** Jie Zhao, Zezhi Ke, Rihua Huang, Xiuyun Wen, Wenbin Liu, Suisui Wang, Xu Zhang, Xiaodong Zhuang, Litao Pan, Lizhen Liao

**Affiliations:** 1 College of Health Science, Guangdong Pharmaceutical University, Guangzhou, Guangdong P.R. China; 2 Guangdong Provincial Key Laboratory of Pharmaceutical Bioactive Substances, Guangdong Pharmaceutical University, Guangzhou, 510006 P. R. China; 3 Department of Cardiology, the First Affiliated Hospital of Sun Yat-Sen University, 58 Zhongshan 2 Road, Guangzhou, 510080 P. R. China; 4 Department of Nuclear Medicine, Guangdong Second Provincial General Hospital, No.466 Road XinGang, Guangzhou, Guangdong 510317 P. R. China; 5 Department of Acupuncture and Massage, Shenzhen Second People ’s Hospital, Shenzhen, 518025 P. R. China

**Keywords:** Physical activity, Age-related diseases, CARDIA study, Mendelian randomization

## Abstract

**Background:**

We aimed to characterize the associations between physical activity levels and the risk of developing age-related diseases in the Coronary Artery Risk Development in Young Adults (CARDIA) study and used Mendelian randomization (MR) to assess whether there are causal relationships between physical activity levels and the risk of developing 8 age-related diseases (coronary atherosclerosis, ischemic heart disease, angina, Alzheimer’s disease, hypertension, type 2 diabetes, hyperlipidemia, and venous thromboembolism).

**Methods:**

Based on the data available in the CARDIA, we obtained data related to five disease states: coronary heart disease, hypertension, diabetes, hyperlipidemia, and venous thromboembolism. Binary logistic regression analysis estimated the multivariable-adjusted associations between different physical activity statuses and diseases. For the MR study, we used summary-level data from a recently published genome-wide association study on physical activity (including vigorous physical activity and accelerometer-based physical activity) conducted with participants from the UK Biobank study. We selected the above 8 age-related diseases as our outcomes.

**Results:**

In the CARDIA-based analysis, the risk of developing coronary heart disease [OR (95% CI): 0.562 (0.397–0.795)], hypertension [OR (95% CI): 0.703 (0.601–0.821)], diabetes [OR (95% CI): 0.783 (0.620–0.988)], and hyperlipidemia [OR (95% CI): 0.792 (0.662–0.949)] was negatively related to physical activity status when participants achieved the physical activity target. Our MR results support a negative causal association between genetically determined vigorous physical activity levels and the risk of developing 3 age-related diseases, namely, angina, hypertension and type 2 diabetes. Moreover, our results also support a negative causal association between genetically determined accelerometer-based physical activity levels and the risk of developing angina.

**Conclusions:**

Promotion of physical activity is likely to prevent specific age-related diseases.

**Supplementary Information:**

The online version contains supplementary material available at 10.1186/s11556-024-00359-2.

## Key message

1. When the amount of physical activity reaches the guidelines, coronary heart disease, hypertension, diabetes, and hyperlipidemia are negatively related to physical activity.

2.Our mendelian randomization results support negative causality between genetically determined vigorous physical activity and 3 age-related diseases, including angina, hypertension, and type 2 diabetes.

3. Our results also support negative causality between genetically determined accelerometer-based physical activity and angina.

**Supplementary Information:**

The online version contains supplementary material available at 10.1186/s11556-024-00359-2.

## Background

Aging is an irreversible and inevitable process and is a risk factor for the pathological progression of diverse age-related diseases [1]. Age-related diseases, including cardiovascular disorders, neurodegenerative diseases, and cancers, are chronic diseases associated with aging. The morbidity of age-related diseases has increased rapidly in the past few decades, and their cost has a substantial impact on social and healthcare expenditures [2].

The relationships between physical activity levels and the risk of developing age-related diseases have been known for many years. A recent meta-analysis including 44 studies and more than 1.5 million participants revealed transparent inverse relationships between moderate and intense physical activity and cardiovascular mortality [3]. Suppose the prevalence of physical inactivity does not change. In that case, 499.2 million new cases of preventable major noncommunicable diseases (most age-related diseases) will occur globally by 2030, and direct health-care costs will reach 520 billion dollars [4]. Given that most epidemiological evidence on the associations between physical activity levels and the risk of developing age-related diseases is based on an observational design, which is less likely to fully account for confounding and reverse causation bias, the causal relationship between physical activity levels and the risk of developing age-related diseases remains unclear. There are two types of physical activity: endurance and resistance training [5]. Physiologically, endurance training refers to exercise in which glucose metabolism depends on oxygen under aerobic conditions. In contrast, resistance training refers to the activity in which force is exerted against weight or overload under anaerobic conditions [6]. These factors may affect age-related diseases differently. Therefore, it is better to discuss them separately.

Mendelian randomization (MR) is an approach that can overcome such limitations by using genetic variants as instrumental variables to evaluate the causal effect of exposure on the outcome [7]. Because genetic variation is randomized among children from the same parents, MR is an increasingly powerful tool for solving problems in epidemiology and human biology [8]. The MR method is less likely to be affected by reverse causality and measurement errors without pleiotropy, making causal inference more feasible compared to conventional study designs.[Jacobs, 1989 #5935].

A recent MR analysis revealed that the presence of 8 age-related diseases (coronary atherosclerosis, ischemic heart disease, angina, Alzheimer’s disease, hypertension, type 2 diabetes, hyperlipidemia, and venous thromboembolism) is causally associated with lower odds of longevity [10]. Here, we chose the above 8 age-related diseases as our primary outcomes. In this study, we aimed to characterize the nature and magnitude of the prospective association between physical activity levels and the risk of developing age-related diseases in the Coronary Artery Risk Development in Young Adults (CARDIA) study. We used MR to assess whether there were causal relationships between physical activity levels and the risk of developing 8 age-related diseases.

## Methods

Physical activity was the exposure, and the onset of 8 age-related diseases (coronary atherosclerosis, ischemic heart disease, angina, Alzheimer’s disease, hypertension, type 2 diabetes, hyperlipidemia, and venous thromboembolism) was chosen as the primary outcome. Our research contains two parts: an association investigation using data from the CARDIA study and an MR study assessing the causal association between physical activity levels and the risk of developing age-related diseases.

The framework flowchart is shown in Supplemental Fig. 1.

### Study population

The CARDIA study was a multicenter, ongoing, longitudinal, prospective cohort study designed to examine the association between cardiovascular health and the risk of developing cardiovascular disease over the life course in a younger population as they aged. Subjects for the study were drawn from four research centers in the United States (Oakland, CA; Minneapolis, MN; Chicago, IL; and Birmingham, AL). A total of 5,115 participants were recruited through the Kaiser Permanente Health Program between 1985 and 1986. Additional relevant details have been previously published [11]. We obtained the data with the permission of the administrator of the CARDIA database (the Coronary Artery Risk Development in Young Adults Study; URLs: https//clinicaltrials.gov/ct2/show/NCT00005130).

Based on the data available in the CARDIA, we obtained data related to five disease states: coronary heart disease, hypertension, diabetes, hyperlipidemia, and venous thromboembolism. Data about the 3 remaining age-related diseases (ischemic heart disease, angina, and Alzheimer’s disease) were unavailable.

First, we excluded participants who had missing total activity scores at baseline (*n* = 4). Second, we excluded participants with missing data for the 5 different disease states (coronary heart disease, hypertension, diabetes mellitus, hyperlipidemia, or venous thromboembolism) at year 25. Finally, we excluded participants with missing covariates. The final participants were *n* = 4935, 3342, 3351, 3250, and 3368, respectively. The specific flow chart of the data inclusion and exclusion criteria is shown in Supplemental Fig. 2.

### Physical activity measurement

The total physical activity score was assessed with the CARDIA Physical Activity History Questionnaire, an interviewer-administered self-report of the frequency of participation in each of 13 categories of sports and exercise during the previous 12 months. The activity duration was acquired by asking participants to report whether they performed at least one hour of exercise in each category during the month. The content, intensity, and time of the activities from the questionnaire were converted through a series of weights to obtain each activity score. The total physical activity score was the sum of 13 activity scores expressed in exercise units (EUs), representing the participant's activity level during the past year. The details of the scoring system have previously been published [12]. For reference, a total activity score of 300 EUs meets the health and human services-recommended target of at least 150 min of moderate-intensity activity per week. In this study, to assess the associations between different physical activity levels and diseases, we divided participants into three groups based on their total activity score at baseline: below physical activity guidelines (< 300 EU), meeting physical activity guidelines (300 ≤ EUs < 900), and meeting three physical activity guidelines (≥ 900 EU).

### Age-related diseases

Coronary heart disease events include hospitalization for acute coronary syndrome or myocardial infarction, exacerbation of symptoms consistent with ischemia but without infarction, or death (fatal myocardial infarction). Blood pressure was measured using a Hawksley randomized zero sphygmomanometer. Blood pressure in the right arm was measured while the participant was sitting, and Korotkoff sounds were recorded for the first and fifth stages. Three measurements were taken, each 5 min apart, and the second and third measurements were finally averaged [13]. Hypertension was defined as the patient being on antihypertensive medication at year 25 or having a systolic blood pressure ≥ 140 mm Hg and a diastolic blood pressure ≥ 90 mm Hg. Diabetes (mainly type 2 diabetes) was defined as the use of glucose-lowering medication at year 25, a fasting blood glucose concentration ≥ 126 mg/dL, a 2-h postprandial blood glucose ≥ 200 mg/dL, or an HbA1c ≥ 6.5%. According to the 2018 Guidelines on the Management of Blood Cholesterol [14], hyperlipidemia is defined as total cholesterol (TC) ≥ 200 mg/dL, triglyceride (TG) ≥ 100 mg/dL, high-density lipoprotein cholesterol (HDL-c) < 40 mg/dL, or low-density lipoprotein cholesterol (LDL-c) ≥ 130 mg/dL. Moreover, nonfatal venous thromboembolic status is reported at year 25 in patients with CARDIA.

### Summary of genome-wide association study (GWAS) data for MR studies

We included summary data from any array-based analysis, including targeted and untargeted arrays, with or without additional imputation for single-nucleotide polymorphisms (SNPs). We also collected data from published GWASs that comprised only the GWAS results that were significant after applying stringent* p*-value thresholds (*P* < 5*10^–8^) using the clumping algorithm (r^2^ threshold = 0.05). The characteristics of the SNPs associated with physical activity levels and their associations with the risk of developing age-related diseases are shown in Supplemental Table 1.

We used summary-level data from a recently published genome-wide association study on physical activity (including vigorous and accelerometer-based physical activity) conducted with participants from the UK Biobank study. [15] For measuring accelerometer-based physical activity (average acceleration), 91,084 participants wore an Axivity-AX3 triaxial accelerometer on their wrist for 7 days [16]. Summary statistics for the physical activity GWAS by Klimentidis et al. [15] are available at https://klimentidis.lab.arizona.edu/content/data. Eight age-related diseases, including coronary atherosclerosis, ischemic heart disease, angina, Alzheimer’s disease, hypertension, type 2 diabetes, hyperlipidemia (low-density lipoprotein cholesterol), and venous thromboembolism, are reported to be causally associated with lower odds of longevity. [10] Our MR analysis selected the above 8 age-related diseases as our outcomes. More details of the studies and datasets used for the analyses are presented in Supplemental Table 2.

### Genetic association analysis by MR

MR can be used to assess the causal effect of exposure on an outcome using genetic variants as instrumental variables [17, 18]. We explored the associations in the following scenarios [19]. (1) Causality: The conventional MR approach (inverse variance weighted, IVW), MR-Egger method, weighted median method, and weighted mode method were used. (1.1) Causality between genetically determined vigorous physical activity levels and the risk of developing 8 age-related diseases was assessed. (1.2) Causality between genetically determined accelerometer-based physical activity levels and the risk of developing 8 age-related diseases. (2) Heterogeneity: To solve the heterogeneity problem, we followed previous researchers’ protocols to determine the final tally of SNPs for inclusion as genetic instruments [20]. (3) Horizontal pleiotropy: A genetic variant is associated with traits on discrete pathways that are also causal for the outcome disease [21]. Unbalanced horizontal pleiotropy distorts the association between the exposure and the outcome, and the effect estimate from the IVW analysis can be exaggerated or diminished. (4) Leave-one-out analysis: To evaluate whether the MR estimate is driven or biased by a single SNP that may have a significant horizontal pleiotropic effect, we re-estimated the effect by sequentially removing one SNP at a time. SNPs that led to a dramatic change in the estimate after their removal can be identified to understand the sensitivity of the estimate to outliers. (5) Funnel plots: Asymmetry in the funnel plot may indicate violations of the assumption through horizontal pleiotropy. [22]

A “causal” relationship is established if the observed association is significant in the IVW analysis with no horizontal pleiotropy.

### Statistical analysis

In the CARDIA-based analysis, binary logistic regression analysis was performed to estimate the multivariable-adjusted associations between different physical activity statuses and diseases using the following models: adjusted for age, sex, smoking status, drinking status, and LDL-c and Cr concentrations at baseline. We expressed the results using odds ratios (ORs) and 95% confidence intervals (95% CIs).

To make the data suitable for MR, we converted odds ratios to log odds ratios and inferred standard errors (SEs) from reported 95% confidence intervals (CIs) from the reported *P* values using the Z distribution. For binary traits, the beta corresponds to the log odds ratio per copy of the effect allele. Beta corresponds to the standard deviation (SD) change in the trait per copy of the effect allele for quantitative traits. The F-statistic is estimated to examine the strength of the genetic instrument for each exposure, and an F-statistic above 10 is considered a sufficiently strong instrument. All the F-statistics in this MR study were above 10. A two-sided *p*-value < 0.05 was considered to indicate statistical significance. All analyses were performed using the TwoSampleMR package [19] (http://app.mrbase.org), IBM SPSS statistics version 26.0 (SPSS, Chicago, IL) and Stata/SE 15.0 (Stata Statistical Software: Release 15. College Station, TX: StataCorp LLC.).

A glossary of terms used in this study is presented in Supplemental Table 3.

## Results

### Associations between physical activity status and disease incidence

In the CARDIA-based analysis, during a follow-up of 25 years, coronary heart disease, hypertension, diabetes, hyperlipidemia, and venous thromboembolism occurred in 165, 1104, 358, 933, and 27 patients, respectively. Table 1 shows the logistic regression analysis results between three physical activity statuses and different diseases. According to the fully adjusted model, the risk of developing coronary heart disease [OR (95% CI): 0.562 (0.397–0.795)], hypertension [OR (95% CI): 0.703 (0.601–0.821)], diabetes [OR (95% CI): 0.783 (0.620–0.988)], and hyperlipidemia [OR (95% CI): 0.792 (0.662–0.949)] were negatively related to physical activity status when participants achieved the recommended physical activity target. Physical activity levels that exceeded the target by three times were negatively associated with the risk of developing hypertension [(OR (95% CI): 0.658 (0.481–0.899)], diabetes [(OR (95% CI): 0.551 (0.320–0.953)] and hyperlipidemia [(OR (95% CI): 0.555 (0.383–0.805)], but not coronary heart disease. However, the risk of developing venous thromboembolism was not associated with physical activity status.

**Table 1:** The relationship between different physical activity statuses and diseases

	Coronary heart disease			Hypertension			Diabetes			Hyperlipidemia			Venous thromboembolism		
Physical activity status	Events/Total n/N	OR (95% CI)	*P* value	Events/Total n/N	OR (95% CI)	*P* value	Events/Total n/N	OR (95% CI)	*P* value	Events/Total n/N	OR (95% CI)	*P* value	Events/Total n/N	OR (95% CI)	*P* value
Below physical activity guidelines	84/2049	Reference	-	529/1379	Reference	-	176/1384	Reference	-	427/1333	Reference	-	11/1391	Reference	-
Meeting physical activity guidelines	61/2504	**0.562 (0.397–0.795)**	**0.001**	508/1719	**0.703 (0.601–0.821)**	** < 0.001**	166/1723	**0.783 (0.620–0.988)**	**0.039**	452/1677	**0.792 (0.662–0.949)**	**0.011**	12/1730	0.989 (0.427–2.288)	0.979
Three times physical activity guidelines	20/382	1.225 (0.719–2.088)	0.455	67/244	**0.658 (0.481–0.899)**	**0.009**	16/244	**0.551 (0.320–0.953)**	**0.033**	54/240	**0.555 (0.383–0.805)**	**0.002**	4/247	2.676 (0.794–9.022)	0.112

Model adjust for age, sex, cigarette smoking status (never, former, current), drinking status (yes, no), low-density lipoprotein cholesterol (LDL-c), Serum creatinine (Cr)

In summary, when participants achieved the physical activity target, they experienced a decreased risk of developing coronary heart disease, hypertension, diabetes, and hyperlipidemia.

### Causality between genetically determined vigorous physical activity levels and the risk of developing 8 age-related diseases

We then used the MR approach to investigate the causal associations between physical activity levels and the risk of developing 8 age-related diseases. Among the 8 age-related diseases, we found that vigorous physical activity decreased the risk of developing angina, hypertension, and type 2 diabetes but not the other 5 age-related diseases. Taking angina as an example (Table 2 and Supplemental Fig. 3), in the IVW analysis, the causal estimate using 7 SNPs as instrumental variables indicated a causal association between genetically determined vigorous physical activity levels and the risk of developing angina (odds ratio [OR]: 0.949; 95% confidence interval [CI]: 0.913–0.987; *P* = 0.009). These results were consistent with the weighted median method (OR, 0.940; 95% CI, 0.900–0.983; *P* = 0.006). The intercept of MR-Egger regression for these 7 SNPs was not statistically significant (*P* = 0.498), indicating no evidence of directional pleiotropy. There was no evidence of heterogeneity between estimates from individual SNPs (*P*_heterogeneity_ = 0.981 [MR-Egger] and *P*_heterogeneity_ = 0.191 [IVW]). In a leave-one-out analysis, we found that no single instrument strongly drove the overall effect of vigorous physical activity levels on the risk of developing angina. In addition, there was no funnel plot asymmetry. The leave-one-out analysis and funnel plot further suggested that no SNPs exhibited horizontal pleiotropy (Supplemental Fig. 3).

**Table 2:** Causality between genetically determined vigorous physical activity and 8 age-related diseases

Trait	Method	nSN**P**	OR	95%（CI）		*P* value	Heterogeneity P	MR-Egger intercept P
Coronary heart disease	MR Egger	7	0.472	0.000	511.532	0.842	0.815	0.959
	Weighted median	7	0.671	0.248	1.819	0.433		
	Inverse variance weighted	7	0.570	0.249	1.304	0.183	0.896	
	Weighted mode	7	0.729	0.188	2.832	0.664		
Ischemic heart disease	MR Egger	7	0.036	0.000	26741.181	0.650	0.005	0.752
	Weighted median	7	0.226	0.055	0.932	0.040		
	Inverse variance weighted	7	0.352	0.070	1.765	0.204	0.009	
	Weighted mode	7	0.293	0.046	1.876	0.243		
Angina	MR Egger	7	0.837	0.595	1.177	0.353	0.164	0.498
	Weighted median	7	0.940	0.900	0.983	0.006		
	**Inverse variance weighted**	**7**	**0.949 **	**0.913 **	**0.987 **	**0.009 **	0.191	
	Weighted mode	7	0.938	0.887	0.992	0.066		
Alzheimer’s disease	MR Egger	6	0.003	0.000	1163635.996	0.600	0.063	0.644
	Weighted median	6	0.535	0.072	3.991	0.542		
	Inverse variance weighted	6	0.479	0.063	3.640	0.477	0.092	
	Weighted mode	6	0.585	0.025	13.546	0.752		
Hypertension	MR Egger	7	41.554	0.001	3025297.525	0.543	0.013	0.397
	Weighted median	7	0.344	0.100	1.180	0.090		
	**Inverse variance weighted**	**7**	**0.220**	**0.053**	**0.918**	**0.038**	0.010	
	Weighted mode	7	0.400	0.094	1.710	0.263		
Type 2 diabetes	MR Egger	7	0.344	0.002	74.838	0.714	0.676	0.937
	Weighted median	7	0.257	0.108	0.609	0.002		
	**Inverse variance weighted**	**7**	**0.275 **	**0.139 **	**0.543 **	**0.000 **	0.788	
	Weighted mode	7	0.226	0.055	0.925	0.084		
High cholesterol	MR Egger	6	599.837	0.316	1137032.745	0.172	0.409	0.658
	Weighted median	6	1.128	0.415	3.068	0.814		
	Inverse variance weighted	6	1.012	0.368	2.788	0.981	0.521	
	Weighted mode	6	1.056	0.225	4.942	0.948		
Venous thromboembolism	MR Egger	7	0.961	0.789	1.170	0.706	0.602	0.838
	Weighted median	7	0.983	0.954	1.013	0.271		
	Inverse variance weighted	7	0.982	0.959	1.005	0.117	0.719	
	Weighted mode	7	0.983	0.938	1.030	0.498		

*SNP* Single-nucleotide polymorphism, *OR* Odds ratio, *CI* Confidence interval

Moreover, vigorous physical activity also decreased the risk of developing hypertension (OR, 0.220; 95% CI, 0.053–0.918; *P* = 0.038) (Table 2 and Supplemental Fig. 4) and type 2 diabetes (OR, 0.275; 95% CI, 0.139–0.543; *P* = 0.000) (Table 2 and Supplemental Fig. 5).

Our MR study revealed a negative causal association between genetically determined vigorous physical activity levels and the risk of developing 3 age-related diseases, including angina, hypertension and type 2 diabetes.

### Causality between genetically determined accelerometer-based physical activity levels and the risk of developing 8 age-related diseases

Among the 8 age-related diseases, we found that accelerometer-based physical activity decreased the risk of developing angina but not the other 7 age-related diseases. As shown in Table 3 and Supplemental Fig. 6, the IVW analysis's causal estimate using 8 SNPs as instrumental variables revealed a causal association between genetically determined accelerometer-based physical activity levels and the risk of developing angina (OR, 0.998; 95% CI, 0.997–1.000; *P* = 0.013). The intercept of MR-Egger regression for these 8 SNPs was not statistically significant (*P* = 0.071), indicating no evidence of directional pleiotropy. There was no evidence of heterogeneity between estimates from individual SNPs (*P*_heterogeneity_ = 0.645 [MR-Egger] and *P*_heterogeneity_ = 0.250 [IVW]). In a leave-one-out analysis, we found that no single instrument strongly drove the overall effect of vigorous physical activity levels on the risk of developing angina. In addition, there was no funnel plot asymmetry. The leave-one-out analysis and funnel plot further suggested that no SNPs exhibited horizontal pleiotropy (Supplemental Fig. 6).

**Table 3:** Causality between genetically determined accelerometer-based physical activity and 8 age-related diseases

Trait	Method	nSNP	OR	95%（CI)		*P* value	Heterogeneity P	MR-Egger intercept P
Coronary heart disease	MR Egger	8	1.196	0.964	1.483	0.154	0.036	0.196
	Weighted median	8	0.995	0.951	1.042	0.842		
	Inverse variance weighted	8	1.024	0.971	1.079	0.382	0.011	
	Weighted mode	8	0.990	0.930	1.055	0.774		
Ischemic heart disease	MR Egger	8	1.008	0.794	1.280	0.950	0.092	0.720
	Weighted median	8	0.956	0.907	1.008	0.096		
	Inverse variance weighted	8	0.964	0.917	1.013	0.148	0.133	
	Weighted mode	8	0.956	0.885	1.032	0.288		
Angina	MR Egger	8	1.004	0.999	1.009	0.190	0.645	0.071
	Weighted median	8	0.999	0.997	1.000	0.098		
	**Inverse variance weighted**	**8**	**0.998 **	**0.997 **	**1.000 **	**0.013 **	0.250	
	Weighted mode	8	0.999	0.996	1.001	0.307		
Alzheimer’s disease	MR Egger	6	1.119	0.642	1.948	0.712	0.000	0.709
	Weighted median	6	0.996	0.905	1.095	0.929		
	Inverse variance weighted	6	1.002	0.882	1.139	0.970	0.001	
	Weighted mode	6	0.893	0.706	1.129	0.387		
Hypertension	MR Egger	8	1.114	0.838	1.480	0.486	0.002	0.344
	Weighted median	8	0.988	0.938	1.042	0.663		
	Inverse variance weighted	8	0.963	0.904	1.026	0.245	0.003	
	Weighted mode	8	0.994	0.924	1.070	0.883		
Type 2 diabetes	MR Egger	6	1.197	0.805	1.779	0.424	0.001	0.422
	Weighted median	6	1.023	0.980	1.067	0.300		
	Inverse variance weighted	6	1.002	0.941	1.065	0.961	0.000	
	Weighted mode	6	1.040	0.983	1.101	0.226		
High cholesterol	MR Egger	6	0.964	0.855	1.086	0.576	0.471	0.938
	Weighted median	6	1.004	0.967	1.042	0.848		
	Inverse variance weighted	6	0.991	0.962	1.022	0.563	0.600	
	Weighted mode	6	1.018	0.957	1.082	0.602		
Venous thromboembolism	MR Egger	8	1.000	0.992	1.007	0.913	0.000	0.938
	Weighted median	8	0.999	0.998	1.001	0.372		
	Inverse variance weighted	8	0.999	0.998	1.001	0.368	0.001	
	Weighted mode	8	0.998	0.995	1.001	0.270		

*SNP* Single-nucleotide polymorphism, *OR* Odds ratio *CI* Confidence interval

In summary, our MR study revealed a negative causal association between genetically determined accelerometer-based physical activity levels and the risk of developing angina.

Table 4 centrally illustrates the associations between physical activity status and the risk of developing 8 age-related diseases from the present epidemiological and Mendelian randomization studies.

**Table 4:** Central illustration about physical activity and 8 age-related diseases from epidemiological and mendelian randomization studies

Outcome	Observational study (association, CARDIA)		Mendelian randomization (SNP causal estimate, GWAS)	
	Meeting physical activity guidelines	Three times physical activity guidelines	Vigorous physical activity	Accelerometer-based physical activity
Coronary heart disease	-	NS	✕	✕
Ischemic heart disease	NA	NA	✕	✕
Angina	NA	NA	**-**	**-**
Alzheimer’s disease	NA	NA	✕	✕
Hypertension	-	-	**-**	✕
Type 2 diabetes	-	-	**-**	✕
High cholesterol	-	-	✕	✕
Venous thromboembolism	NS	NS	✕	✕

-: negative associated, *NA* not available, *NS* No statistical difference

✕: no causality; **-**: negative causality

## Discussion

The CARDIA-based analysis showed that achieving the recommended physical activity target decreased the risk of developing coronary heart disease, hypertension, diabetes, and hyperlipidemia. Furthermore, our MR results support a negative causal relationship between genetically determined vigorous physical activity levels and the risk of developing 3 age-related diseases (angina, hypertension, and type 2 diabetes). Our results also revealed a negative causal association between genetically determined accelerometer-based physical activity levels and the risk of developing angina. As such, physical activity may serve as an excellent prognostic factor for some age-related diseases and is likely to prevent specific age-related diseases (Table 4).

### Age-related diseases

The aging of the population is a worldwide phenomenon. It is a global occurrence, with the proportion of people aged 65 years or older worldwide increasing from 9.3% in 2020 to 22.6% by 2100. [23] High incidences of morbidity and mortality associated with age-related diseases among the elderly population are a socioeconomic challenge. Due to the increasing aging population and the increasing prevalence of age-related diseases, it is essential to develop novel preventive and therapeutic interventions to reduce the burden of age-related diseases. Notably, a recent MR analysis revealed that out of 4587 environmental exposures, the presence of 8 age-related diseases, including coronary atherosclerosis, ischemic heart disease, angina, Alzheimer’s disease, hypertension, type 2 diabetes, hyperlipidemia, and venous thromboembolism, is causally associated with a greater risk of premature mortality [10]. Genetically speaking, preventing the above 8 age-related diseases can improve healthy longevity and help mitigate the costs of an aging society.

### Physical activity levels and the risk of developing age-related diseases

Promoting physical activity is one of the crucial methods for improving the general population's quality of life throughout the lifespan. The health benefits of physical activity are well recognized and observed across multiple organ systems. These beneficial effects enhance overall resilience, health span and longevity [24]. In 2020, the WHO stated that all adults should engage in 150–300 min of moderate-intensity physical activity per week, 75–150 min of vigorous-intensity physical activity per week or an equivalent combination of moderate-intensity and vigorous-intensity physical activity per week. [25] However, approximately one-third of adults worldwide do not meet the minimal intensity or duration of physical activity recommended by the WHO. Physical inactivity has become a significant public health threat and is associated with increased mortality [26] and a considerable economic burden [27].

For patients, physical activity has also been prescribed as a medicine for different age-related diseases. [28] An increasing number of cohort studies, systematic reviews, and meta-analyses have documented the beneficial effects of physical activity in reducing cardiovascular risk factors and the risk of cardiovascular events [29–31]. In clinical interventions, appropriate exercise training has enhanced exercise capacity and cardiorespiratory fitness, reduced hospitalisation, and improved quality of life in patients with hypertension, coronary heart disease, cardiomyopathy, and heart failure [32]. The above epidemiological evidence indicates a link between physical activity levels and many age-related diseases.

### Epidemiological evidence of the link between physical activity levels and the risk of developing 8 age-related diseases

In the CARDIA-based analysis, we divided participants into three groups based on their total activity score at baseline: below physical activity guidelines, meeting physical activity guidelines, and meeting three physical activity guidelines. For MR analysis, we chose vigorous physical activity to represent the resistance exercise level and accelerometer-based physical activity to represent the endurance exercise level.

Previously, in the CARDIA-based analysis, a lower physical activity score (per 100 units) at 18 years of age was associated with a 4% greater risk of developing hypertension. Each additional 1-unit reduction per year in physical activity score is associated with 2% greater annual risk of developing hypertension [33]. After adjusting for age, sex, race, baseline smoking status, systolic blood pressure, alcohol intake, high-density lipoprotein cholesterol, dietary fiber, dietary sodium, fasting glucose and body mass index, physical activity was still inversely associated with incident hypertension [34]. Moreover, lower physical activity scores in 18-year-olds are associated with greater odds of premature coronary heart disease [35]. Accelerometer-determined moderate-vigorous physical activity is associated with a 37% to 67% decreased risk of incident type 2 diabetes in a dose–response relationship [36].

In the CARDIA-based analysis, our results indicate that achieving the recommended physical activity target was associated with a decreased risk of developing coronary heart disease, hypertension, diabetes, and hyperlipidemia, which are negatively related to physical activity (Table 1). Therefore, we have added more convincing evidence supporting an association between physical activity levels and the risk of developing age-related diseases from young adulthood to middle age.

### Comparison with previous MR studies

MR studies the causal effects of modifiable exposures (i.e., potential risk factors) on health, social, and economic outcomes using genetic variants associated with the specific exposures of interest [9]. Our study used the MR approach to investigate the causal associations between physical activity levels and the risk of developing 8 age-related diseases.

### Physical activity levels and the risk of developing heart diseases (coronary heart disease, ischemic heart disease, angina)

Cardiovascular disease increases the burden on public health systems, especially in older adults, mainly because this group of patients frequently suffers from multiple comorbidities. The beneficial effects of physical activity on the cardiovascular system have been extensively reported [37, 38]. However, our MR study did not reveal a causal association between genetically determined physical activity levels and the risk of developing 2 kinds of heart disease (coronary heart disease and ischemic heart disease) (Tables 2 and 3). In line with our findings, Martin Bahls et al. [39] reported no causal associations between genetically predicted self-reported moderate to vigorous physical activity, accelerometer-based physical activity or an accelerometer fraction of accelerations > 425 milligravities or sedentary behaviour and the risk of developing coronary artery disease. Similarly, Chengui Zhuo et al [40]. also demonstrated that no causal effect was found between physical activity levels and the risk of developing coronary heart disease. Moreover, Doherty et al [41]. recently performed a one-sample MR analysis, and no associations were observed between physical activity or walking status and the risk of developing coronary heart disease.

In contrast, Zhuang et al. [42] provided suggestive evidence that vigorous physical activity levels decreased the risk of developing coronary heart disease. The definitions and assessments of physical activity may explain the differences between our findings and those of Zhuang. Based on our MR results and those of other MR studies, previous observational studies may have been biased, and physical activity levels may not be causally related to the risk of developing coronary heart disease or ischemic heart disease.

For angina, to the best of our knowledge, the present study may be the first to assess physical activity as a protective factor against the development of angina by using the MR approach (Tables 2 and 3, Supplemental Figs. 3 and 6). Typical angina or angina pectoris is a symptom of myocardial ischemia. It is characterized by chest discomfort or anginal equivalent provoked with exertion and alleviated at rest or with nitroglycerin. It is often one of the first manifestations or warning signs of underlying coronary disease [43]. Although our present MR results do not support a causal association between genetically determined physical activity levels and the risk of developing coronary heart disease, physical activity is still recommended for angina control.

### Physical activity levels and the risk of developing Alzheimer’s disease

Alzheimer's disease is the most severe age-related neurodegenerative disease and causes destructive and irreversible cognitive decline [44]. Previous meta-analyses have indicated that physical activity intervention significantly improves the cognition of patients diagnosed with Alzheimer's disease or slows the decrease in cognition [28, 45, 46]. A recent systematic review and meta-analysis revealed that physical activity is associated with a lower incidence of all-cause dementia and Alzheimer's disease, even at longer follow-ups, suggesting that physical activity is a modifiable protective lifestyle factor, even after reducing the effects of reverse causation [47]. However, our MR study did not reveal a causal association between genetically determined physical activity levels and the risk of developing Alzheimer’s disease (Tables 2 and 3). Similarly, 3 previous MR studies from other researchers also indicated a noncausal association between physical activity levels and the risk of developing Alzheimer's disease [48–50]. Moreover, an MR study proved that genetically predicted walking (not an overall activity, sedentary behaviour, or moderate-intensity activity) might be associated with a reduced risk of developing Alzheimer's disease [51].

In summary, the effect of physical activity on the risk of developing Alzheimer's disease is still unclear. When exploring the causal association between physical activity levels and the risk of developing Alzheimer's disease, it is better to divide physical activity into more specific types, such as walking, jogging, or swimming.

### Physical activity levels and the risk of developing hypertension

Hypertension is a significant risk factor for cardiovascular disease [52] and typically coexists with other cardiovascular disease risk factors and unhealthy lifestyle behaviours, such as physical inactivity [53]. A randomized controlled trial revealed that exercise training lowers blood pressure and is causally related to hypertension [54]. Our MR study revealed a negative causal association between genetically determined vigorous physical activity levels (but not between accelerometer-based physical activity) and the risk of developing hypertension (Tables 2 and 3). In contrast, Sabine van Oort et al. [55]. reported insufficient evidence for a causal relationship between physical activity levels and the risk of developing hypertension. This inconsistency may be due to using different datasets for hypertension MR analyses. Sabine van Oort et al. [55]. extracted data on the genetic associations between the instrumental variables and the risk of developing hypertension from 2 European cohorts: the FinnGen Study (https://finngen.gitbook.io/finngen-documentation/-LvQ4yR2YFUM5eFTjieO./Accessed February 20, 2020) and the UK Biobank (https://www.ukbiobank.ac.uk/wp-content/uploads/2011/11/UK-Biobank-Protocol.pdf. Accessed August 2020). We chose a more recent dataset (from 2021) for hypertension analyses on the MR-based platform (ID: finn-b-I9_HYPTENSESS).

### Physical activity levels and the risk of developing type 2 diabetes

Type 2 diabetes is one of the major chronic diseases accounting for a substantial proportion of the disease burden worldwide. Previous randomized controlled trials and reviews have shown a protective association between physical activity levels and type 2 diabetes outcomes [56, 57]. Our MR study revealed a negative causal relationship between genetically determined vigorous physical activity levels (but not accelerometer-based physical activity) and the risk of developing type 2 diabetes (Tables 2 and 3). In line with our results, Vanesa Bellou et al. [58] reported that decreased physical activity, high sedentary time and duration of television watching presented robust evidence for an increased risk of type 2 diabetes. Moreover, a prospective study in the UK Biobank revealed that a healthy lifestyle, including regular physical activity, is associated with a lower risk of all-cause mortality and mortality due to cardiovascular disease, cancer, respiratory disease, and digestive disease among individuals with type 2 diabetes [59]. However, Christa Meisinger et al. [60] reported that genetically predicted objectively measured average or vigorous physical activity and sedentary behaviour levels are not associated with type 2 diabetes risk in the general population. We analyzed the different conclusions that may come from the diverse strategies used for selecting instrumental variables for physical activity.

### Physical activity levels and the risk of developing hyperlipidemia

For hyperlipidemia, we chose LDL cholesterol to represent the cholesterol level. Our MR study did not support a causal association between genetically determined vigorous physical activity levels and the risk of developing hyperlipidemia (Tables 2 and 3). Similar to our results, Zhenhuang Zhuang et al. [42] also demonstrated that genetically predicted physical activity levels do not significantly influence lipid levels.

### Physical activity levels and the risk of developing venous thromboembolism

The present study may be the first to investigate the causal relationship between physical activity levels and the risk of developing venous thromboembolism (Tables 2 and 3). However, although venous thromboembolism status is causally associated with lower odds of longevity [10], our results do not support a causal association between genetically determined physical activity levels and the risk of developing venous thromboembolism.

### Limitations

First, there are many other confounders that we cannot adjust since they were not measured in the CARDIA study at baseline, such as many inflammatory markers. Second, the physical activity measurement could be better in the CARDIA cohort. The Physical Activity History Questionnaire collected data on the total months the participants were involved in 13 different intensities and categories of activity in the past year. As a result, physical activity measurements need to be more objective. Third, given the limited power of the MR analysis (with SNPs explaining 1–3% of physical activity), there is not enough strength to support the causal effect between physical activity levels and some kinds of age-related diseases. However, this does not mean that causal impact does not exist. Further large-scale studies or longitudinal studies are required to validate the true associations.

## Conclusions

In conclusion, achieving the recommended physical activity target decreased the risk of developing coronary heart disease, hypertension, diabetes, and hyperlipidemia. Our results support a negative causal relationship between genetically determined vigorous physical activity levels and the risk of developing 3 age-related diseases, namely, angina, hypertension, and type 2 diabetes. Our results also support a negative causal association between genetically determined accelerometer-based physical activity levels and the risk of developing angina. Precise and individualized prescriptions of physical activity should be provided to older adults who are susceptible to specific age-related diseases.

## Supplementary Information

Supplementary Material 1: Supplemental Fig. 1 The framework flowchart of this study. Comparison of observational and Mendelian randomization studies to help understand the causal associations between physical activity levels and the risk of developing age-related diseases. SNP, single nucleotide polymorphism; GWAS, genome-wide association study.

Supplementary Material 2: Supplemental Fig. 2 Flow chart of the inclusion and exclusion criteria used in the CARDIA study. CARDIA, Coronary Artery Risk Development in Young Adults.

Supplementary Material 3: Supplemental Fig. 3 Mendelian randomization study of the effect of vigorous physical activity levels on the risk of developing angina. (A) Forest plots of the causal effects of vigorous physical activity levels on the risk of developing angina. The red points show the combined causal estimate using all SNPs in a single instrument and two methods (MR-Egger and inverse-variance weighted). The horizontal lines denote the 95% confidence intervals. (B) Scatter plots. The slopes of each line in the scatter plot represent the causal association for each method. (C) Leave-one-out sensitivity analysis. (D) Funnel plots. MR, Mendelian randomization; SNP, single-nucleotide polymorphism; inverse‐variance weighted.

Supplementary Material 4: Supplemental Fig. 4 Mendelian randomization study of the effect of vigorous physical activity levels on the risk of developing hypertension. Forest plots of the causal effects of vigorous physical activity levels on the risk of developing hypertension. (B) Scatter plots. (C) Leave-one-out sensitivity analysis. (D) Funnel plots. MR, Mendelian randomization; SNP, single-nucleotide polymorphism; IVW, inverse‐variance weighted.

Supplementary Material 5: Supplemental Fig. 5 Mendelian randomization study of the effect of vigorous physical activity levels on the risk of developing type 2 diabetes. (A) Forest plots of the causal effects of vigorous physical activity levels on the risk of developing type 2 diabetes. (B) Scatter plots. (C) Leave-one-out sensitivity analysis. (D) Funnel plots. MR, Mendelian randomization; SNP, single-nucleotide polymorphism; IVW, inverse‐variance weighted.

Supplementary Material 6: Figure Supplemental 6 Mendelian randomization study of the effect of accelerometer-based physical activity levels on the risk of developing angina. (A) Forest plots of the causal effects of accelerometer-based physical activity levels on the risk of developing angina. (B) Scatter plots. (C) Leave-one-out sensitivity analysis. (D) Funnel plots. MR, Mendelian randomization; SNP, single-nucleotide polymorphism; IVW, inverse‐variance weighted.

Supplementary Material 7.

Supplementary Material 8.

Supplementary Material 9.

## Data Availability

The datasets used during the current study available from the corresponding author on reasonable request.

## Abbreviations

GWAS: Genome-wide association study
CARDIA: Coronary Artery Risk Development in Young Adults
MR: Mendelian randomization
EUs: Exercise units
TC: Total cholesterol
TG: Triglycerides
HDL-c: High-density lipoprotein cholesterol
LDL-c: Low-density lipoprotein cholesterol
SNP: Single nucleotide polymorphism
IVW: Inverse variance weighted
OR: Odds ratio
95% CI: 95% Confidence interval
SE: Standard error
SD: Standard deviation
