# Supplementary figures and images for: Physical activity and the risk of developing 8 age-related diseases: epidemiological and Mendelian randomization studies
Source: Eur Rev Aging Phys Act. 2024 Sep 18;21:24. doi: 10.1186/s11556-024-00359-2 (PMC11412029; doi:10.1186/s11556-024-00359-2)

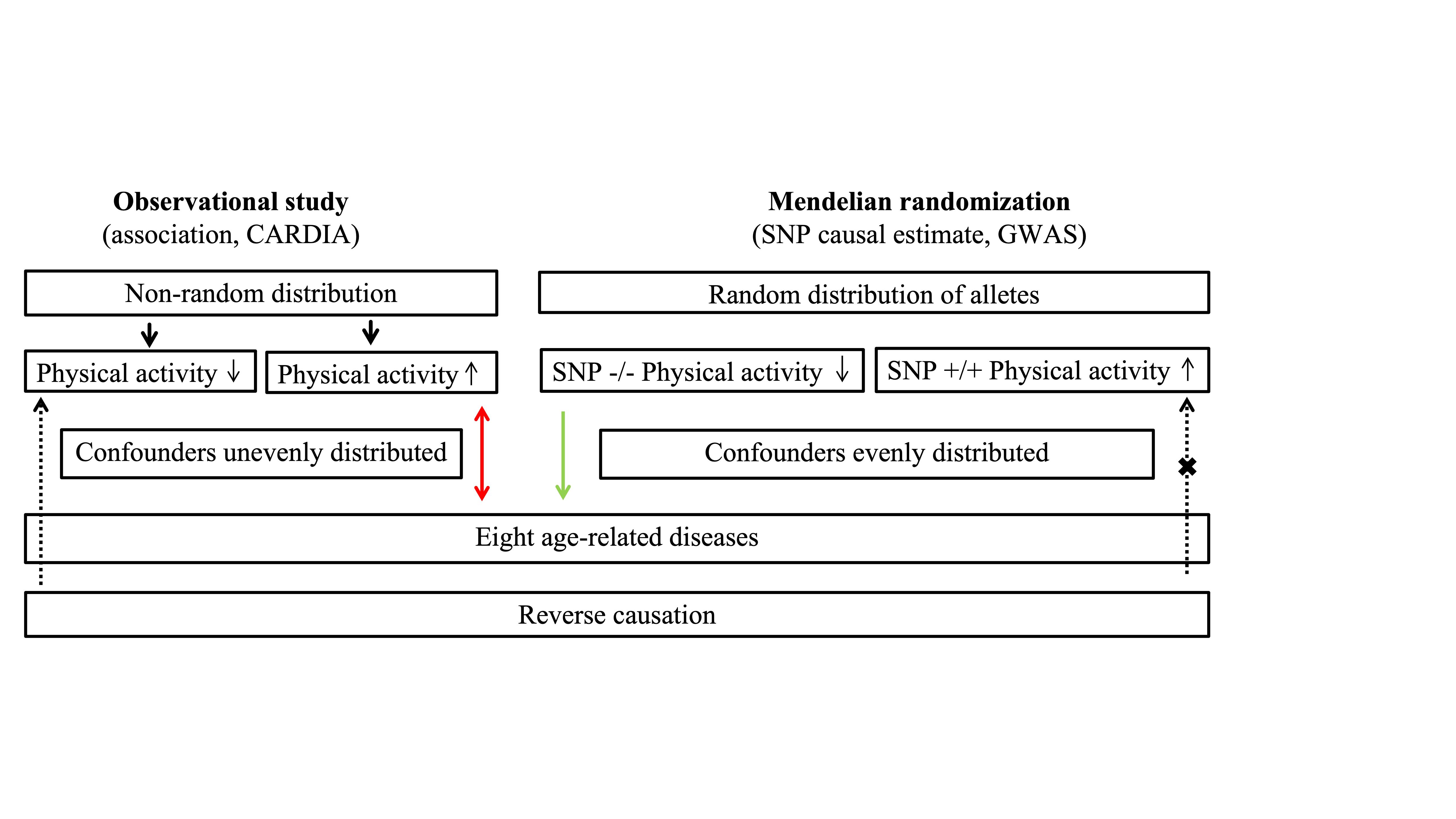

Supplement: Supplementary file 1 — Supplementary Material 1: Supplemental Fig. 1 The framework flowchart of this study. Comparison of observational and Mendelian randomization studies to help understand the causal associations between physical activity levels and the risk of developing age-related diseases. SNP, single nucleotide polymorphism; GWAS, genome-wide association study. [file 11556_2024_359_MOESM1_ESM.jpg]

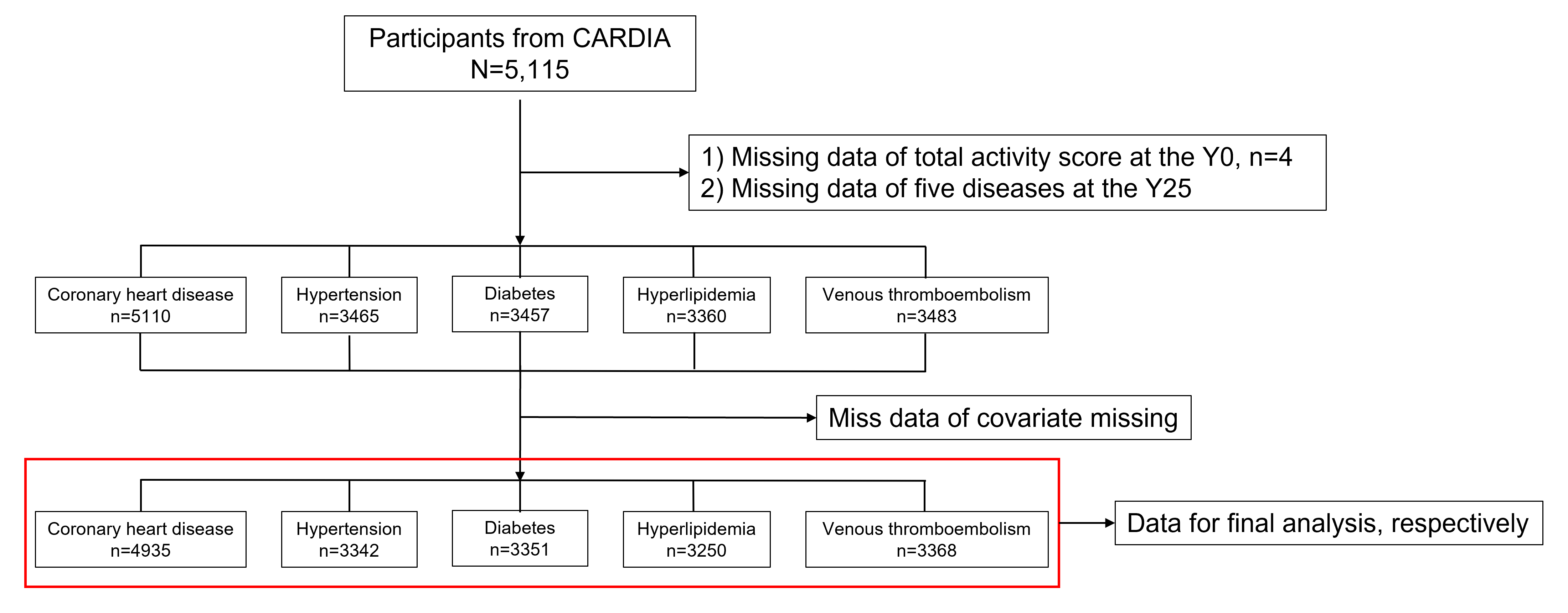

Supplement: Supplementary file 2 — Supplementary Material 2: Supplemental Fig. 2 Flow chart of the inclusion and exclusion criteria used in the CARDIA study. CARDIA, Coronary Artery Risk Development in Young Adults. [file 11556_2024_359_MOESM2_ESM.tif]

A

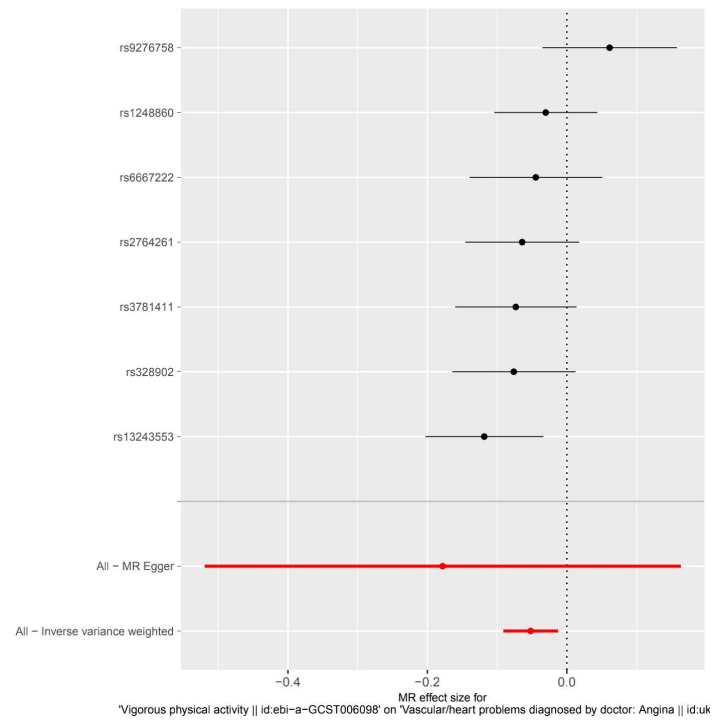

B

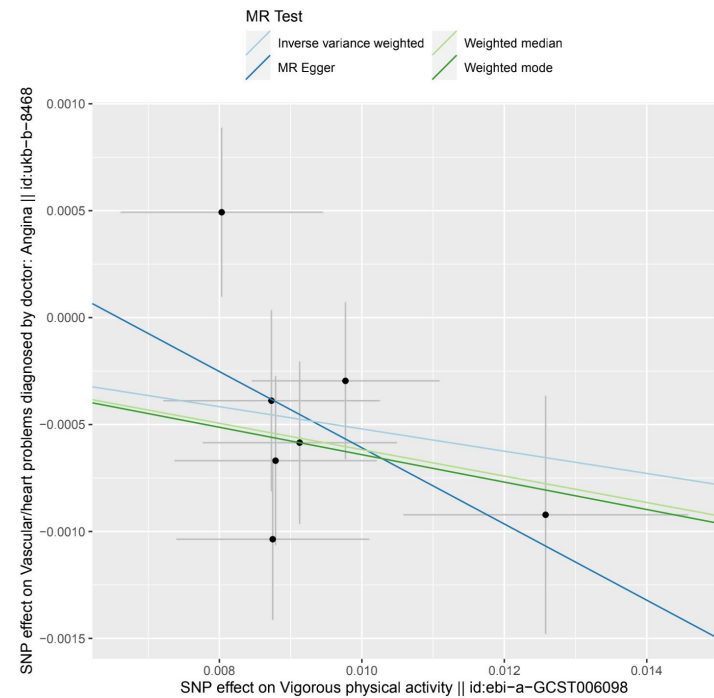

C

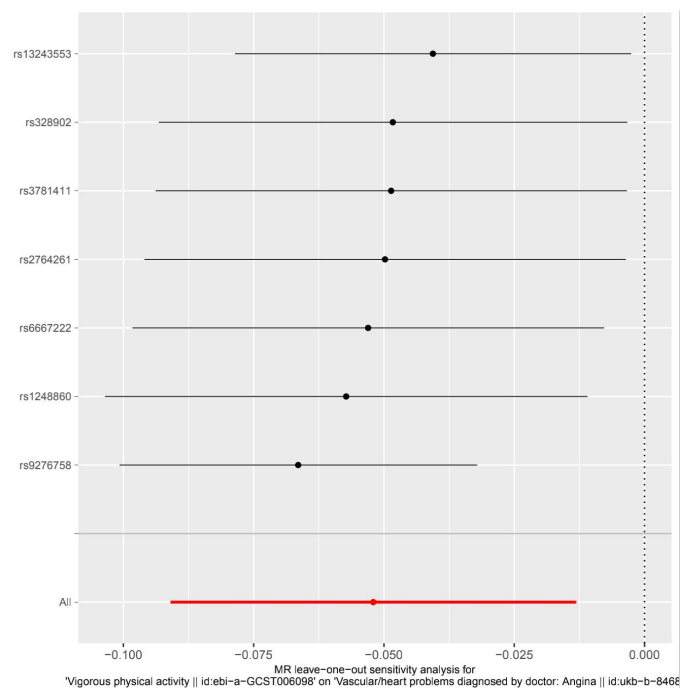

D

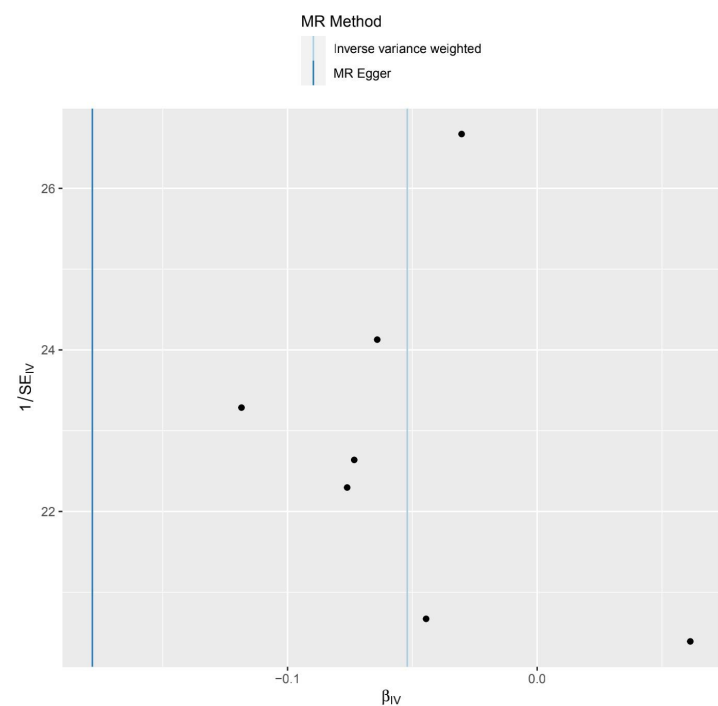

Supplement: Supplementary file 3 — Supplementary Material 3: Supplemental Fig. 3 Mendelian randomization study of the effect of vigorous physical activity levels on the risk of developing angina. (A) Forest plots of the causal effects of vigorous physical activity levels on the risk of developing angina. The red points show the combined causal estimate using all SNPs in a single instrument and two methods (MR-Egger and inverse-variance weighted). The horizontal lines denote the 95% confidence intervals. (B) Scatter plots. The slopes of each line in the scatter plot represent the causal association for each method. (C) Leave-one-out sensitivity analysis. (D) Funnel plots. MR, Mendelian randomization; SNP, single-nucleotide polymorphism; inverse‐variance weighted. [file 11556_2024_359_MOESM3_ESM.pdf]

A

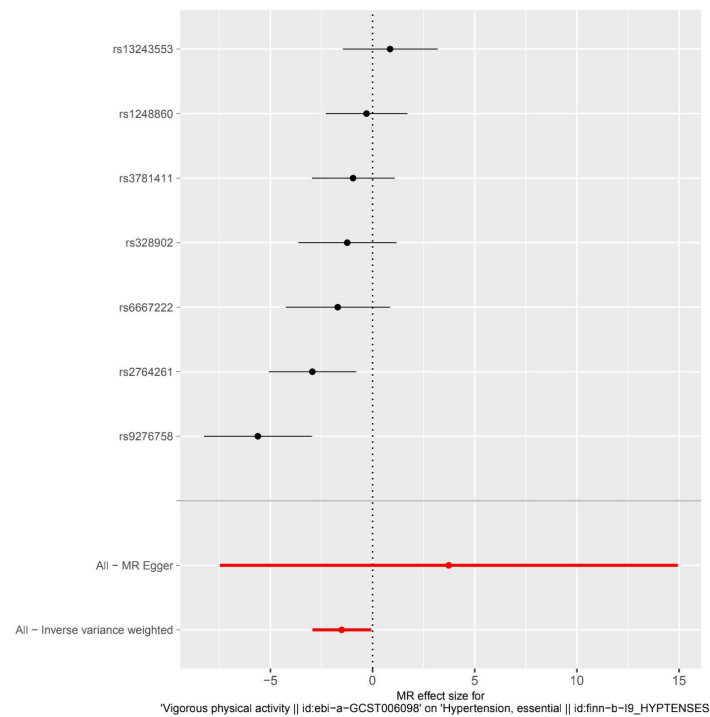

B

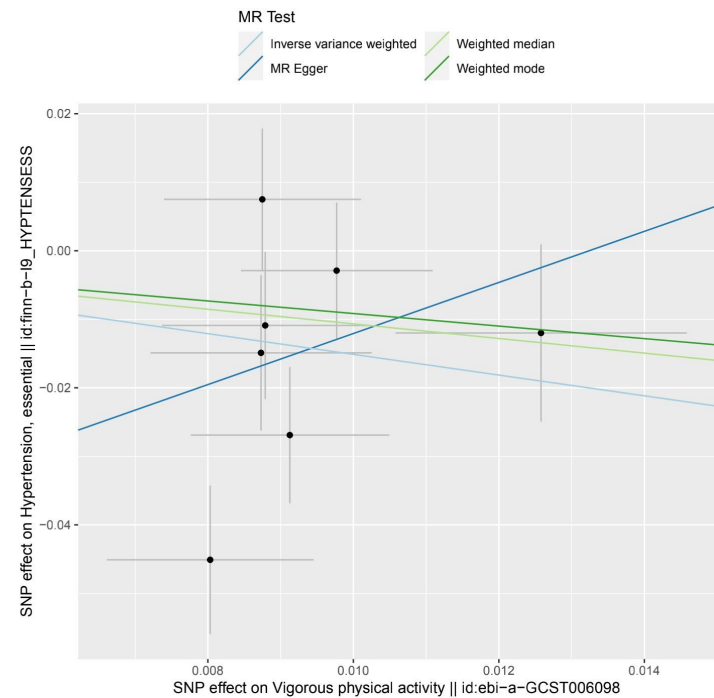

C

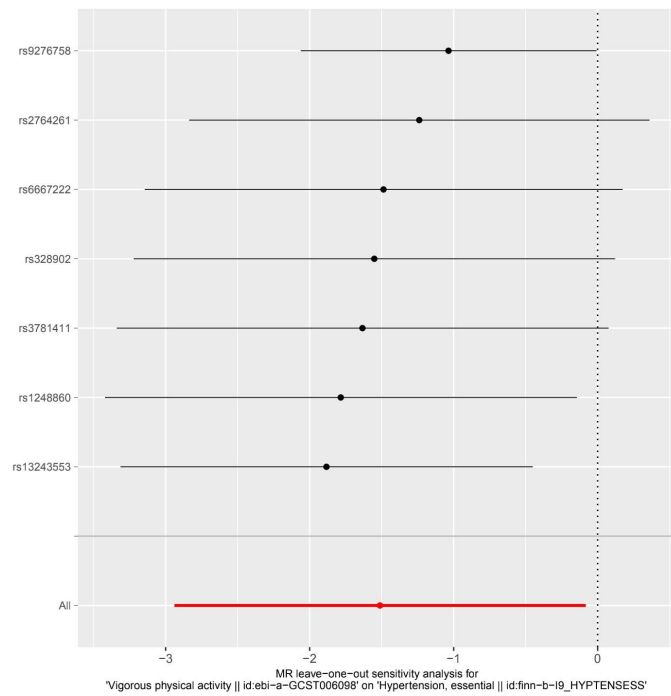

D

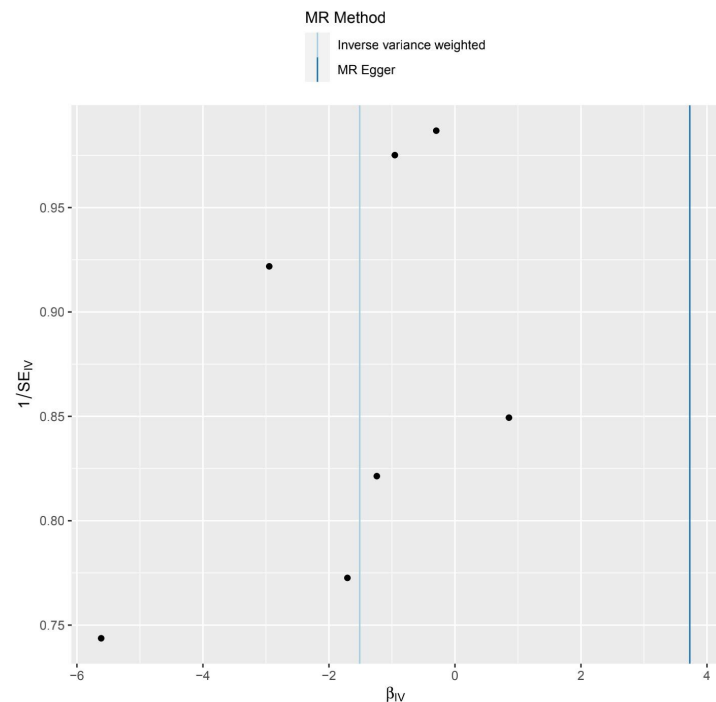

Supplement: Supplementary file 4 — Supplementary Material 4: Supplemental Fig. 4 Mendelian randomization study of the effect of vigorous physical activity levels on the risk of developing hypertension. Forest plots of the causal effects of vigorous physical activity levels on the risk of developing hypertension. (B) Scatter plots. (C) Leave-one-out sensitivity analysis. (D) Funnel plots. MR, Mendelian randomization; SNP, single-nucleotide polymorphism; IVW, inverse‐variance weighted. [file 11556_2024_359_MOESM4_ESM.pdf]

A

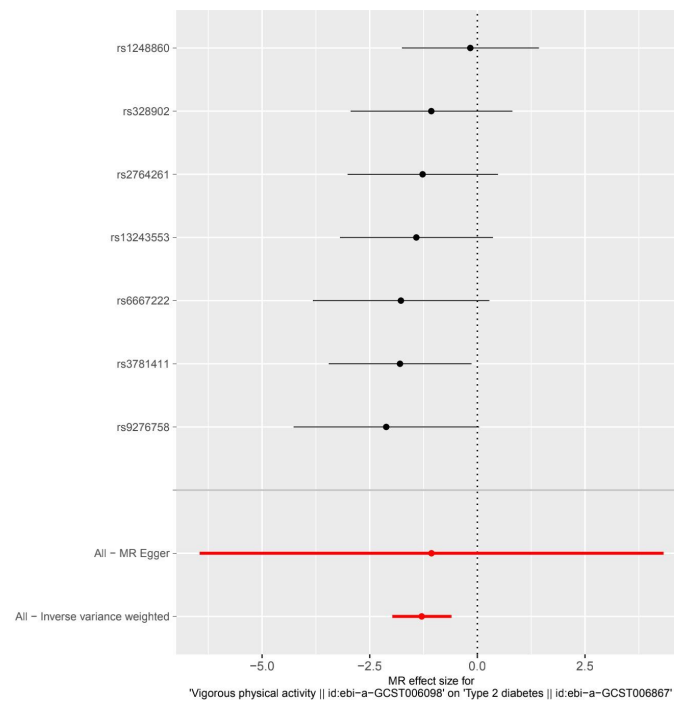

B

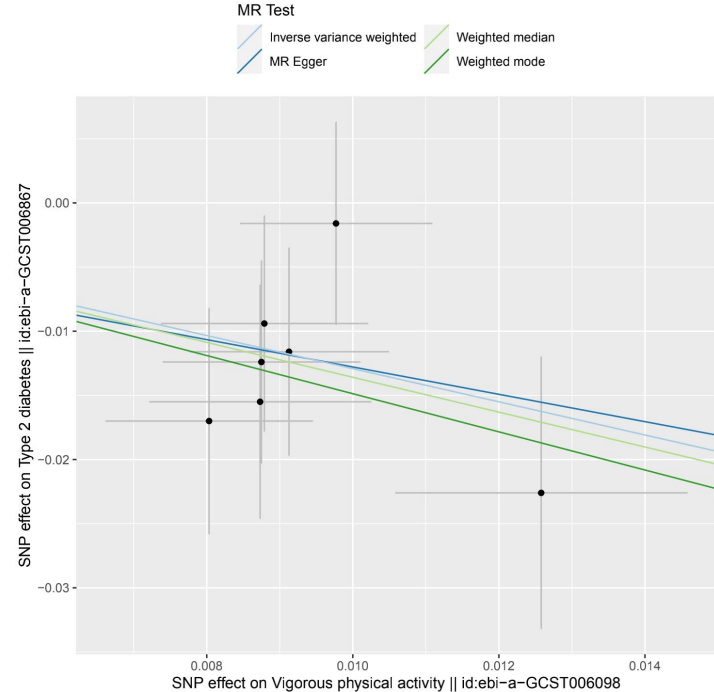

C

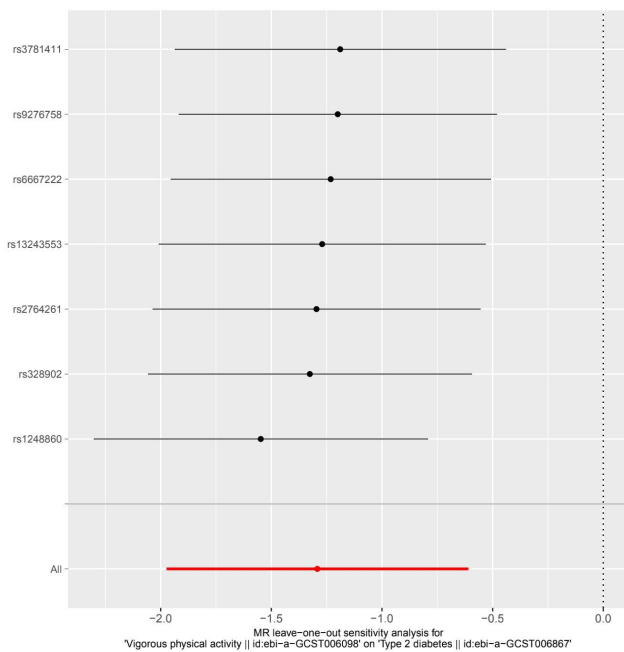

D

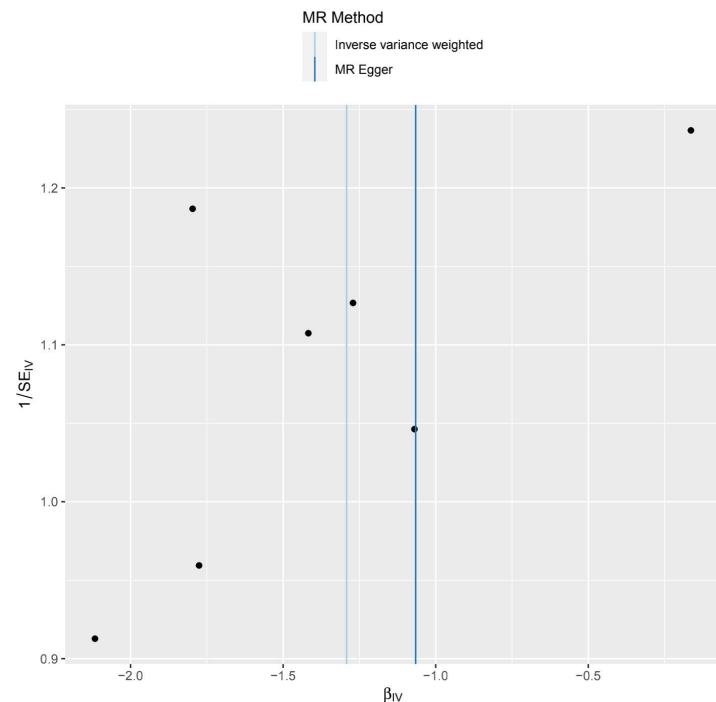

Supplement: Supplementary file 5 — Supplementary Material 5: Supplemental Fig. 5 Mendelian randomization study of the effect of vigorous physical activity levels on the risk of developing type 2 diabetes. (A) Forest plots of the causal effects of vigorous physical activity levels on the risk of developing type 2 diabetes. (B) Scatter plots. (C) Leave-one-out sensitivity analysis. (D) Funnel plots. MR, Mendelian randomization; SNP, single-nucleotide polymorphism; IVW, inverse‐variance weighted. [file 11556_2024_359_MOESM5_ESM.pdf]

A

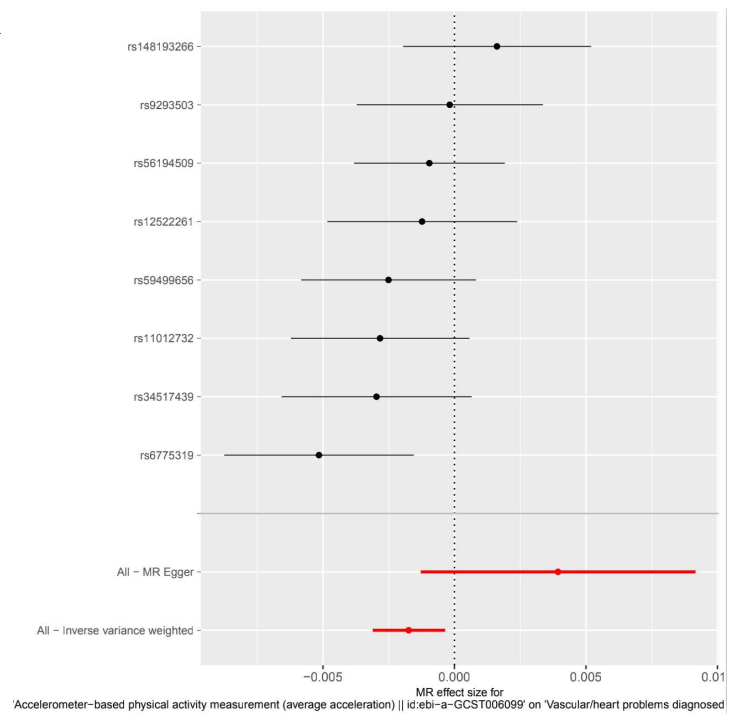

B

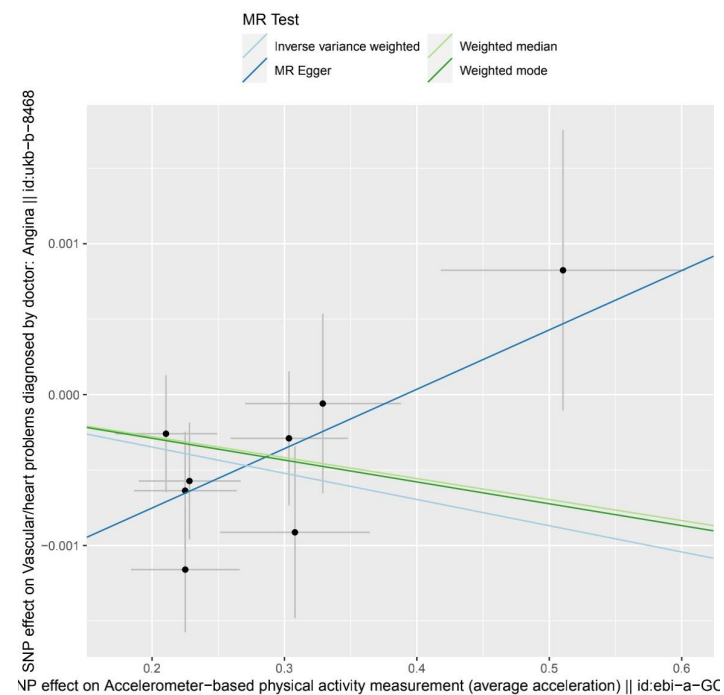

C

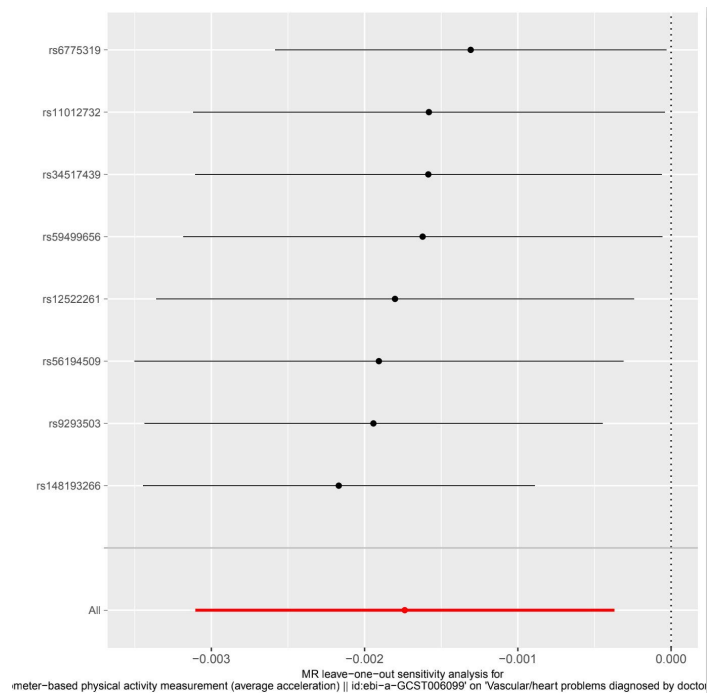

D

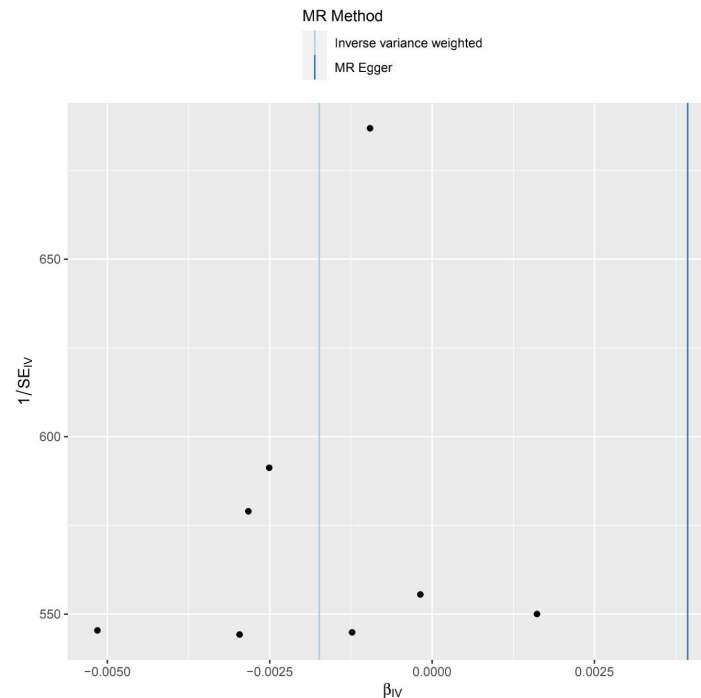

Supplement: Supplementary file 6 — Supplementary Material 6: Figure Supplemental 6 Mendelian randomization study of the effect of accelerometer-based physical activity levels on the risk of developing angina. (A) Forest plots of the causal effects of accelerometer-based physical activity levels on the risk of developing angina. (B) Scatter plots. (C) Leave-one-out sensitivity analysis. (D) Funnel plots. MR, Mendelian randomization; SNP, single-nucleotide polymorphism; IVW, inverse‐variance weighted. [file 11556_2024_359_MOESM6_ESM.pdf]
