# Supplementary material for: Physical activity and the risk of developing 8 age-related diseases: epidemiological and Mendelian randomization studies
Source: Eur Rev Aging Phys Act. 2024 Sep 18;21:24. doi: 10.1186/s11556-024-00359-2 (PMC11412029; doi:10.1186/s11556-024-00359-2)
Supplement: Supplementary file 7 — Supplementary Material 7. [file 11556_2024_359_MOESM7_ESM.doc]

Supplemental Table 1 Characteristics of the SNPs associated with vigorous physical activity/ ccelerometer-based physical activity and eight age-related diseases

| SNP | effect_allele.exposure | effect_allele.outcome | eaf.exposure | eaf.outcome | Associations with vigorous physical activity | | | Associations with coronary heart disease | | |
| --- | --- | --- | --- | --- | --- | --- | --- | --- | --- | --- |
|  |  |  |  |  | Beta | SE | P | Beta | SE | P |
| rs1248860 | A | A | 0.516 | 0.514 | 0.010 | 0.001 | 0.000 | -0.002 | 0.009 | 0.832 |
| rs13243553 | A | A | 0.392 | 0.425 | -0.009 | 0.001 | 0.000 | 0.006 | 0.009 | 0.498 |
| rs2764261 | G | G | 0.626 | 0.574 | -0.009 | 0.001 | 0.000 | 0.002 | 0.010 | 0.850 |
| rs328902 | T | T | 0.315 | 0.287 | 0.009 | 0.001 | 0.000 | -0.017 | 0.010 | 0.096 |
| rs3781411 | T | T | 0.124 | 0.124 | -0.013 | 0.002 | 0.000 | 0.010 | 0.014 | 0.476 |
| rs6667222 | C | C | 0.252 | 0.238 | -0.009 | 0.002 | 0.000 | -0.003 | 0.011 | 0.783 |
| rs9276758 | A | A | 0.312 | 0.336 | -0.008 | 0.001 | 0.000 | 0.004 | 0.010 | 0.726 |
| SNP | effect_allele.exposure | effect_allele.outcome | eaf.exposure | eaf.outcome | Associations with vigorous physical activity | | | Associations with ischemic heart disease | | |
|  |  |  |  |  | Beta | SE | P | Beta | SE | P |
| rs1248860 | A | A | 0.516 | 0.434 | 0.010 | 0.001 | 0.000 | -0.014 | 0.011 | 0.198 |
| rs13243553 | A | A | 0.392 | 0.355 | -0.009 | 0.001 | 0.000 | -0.003 | 0.012 | 0.830 |
| rs2764261 | G | G | 0.626 | 0.578 | -0.009 | 0.001 | 0.000 | 0.016 | 0.011 | 0.159 |
| rs328902 | T | T | 0.315 | 0.302 | 0.009 | 0.001 | 0.000 | 0.007 | 0.012 | 0.584 |
| rs3781411 | T | T | 0.124 | 0.176 | -0.013 | 0.002 | 0.000 | 0.027 | 0.015 | 0.060 |
| rs6667222 | C | C | 0.252 | 0.251 | -0.009 | 0.002 | 0.000 | -0.022 | 0.013 | 0.090 |
| rs9276758 | A | A | 0.312 | 0.346 | -0.008 | 0.001 | 0.000 | 0.041 | 0.012 | 0.001 |
| SNP | effect_allele.exposure | effect_allele.outcome | eaf.exposure | eaf.outcome | Associations with vigorous physical activity | | | Associations with angina | | |
|  |  |  |  |  | Beta | SE | P | Beta | SE | P |
| rs1248860 | A | A | 0.516 | 0.515 | 0.010 | 0.001 | 0.000 | 0.000 | 0.000 | 0.420 |
| rs13243553 | A | A | 0.392 | 0.391 | -0.009 | 0.001 | 0.000 | 0.001 | 0.000 | 0.006 |
| rs2764261 | G | G | 0.626 | 0.626 | -0.009 | 0.001 | 0.000 | 0.001 | 0.000 | 0.120 |
| rs328902 | T | T | 0.315 | 0.315 | 0.009 | 0.001 | 0.000 | -0.001 | 0.000 | 0.090 |
| rs3781411 | T | T | 0.124 | 0.124 | -0.013 | 0.002 | 0.000 | 0.001 | 0.001 | 0.097 |
| rs6667222 | C | C | 0.252 | 0.251 | -0.009 | 0.002 | 0.000 | 0.000 | 0.000 | 0.360 |
| rs9276758 | A | A | 0.312 | 0.312 | -0.008 | 0.001 | 0.000 | 0.000 | 0.000 | 0.210 |
| SNP | effect_allele.exposure | effect_allele.outcome | eaf.exposure | eaf.outcome | Associations with vigorous physical activity | | | Associations with Alzheimer’s disease | | |
|  |  |  |  |  | Beta | SE | P | Beta | SE | P |
| rs1248860 | A | A | 0.516 | NA | 0.010 | 0.001 | 0.000 | -0.013 | 0.016 | 0.394 |
| rs13243553 | A | A | 0.392 | NA | -0.009 | 0.001 | 0.000 | -0.018 | 0.016 | 0.251 |
| rs2764261 | G | G | 0.626 | NA | -0.009 | 0.001 | 0.000 | 0.045 | 0.016 | 0.005 |
| rs328902 | T | T | 0.315 | NA | 0.009 | 0.001 | 0.000 | 0.010 | 0.017 | 0.568 |
| rs3781411 | T | T | 0.124 | NA | -0.013 | 0.002 | 0.000 | 0.013 | 0.024 | 0.595 |
| rs6667222 | C | C | 0.252 | NA | -0.009 | 0.002 | 0.000 | -0.005 | 0.019 | 0.781 |
| SNP | effect_allele.exposure | effect_allele.outcome | eaf.exposure | eaf.outcome | Associations with vigorous physical activity | | | Associations with hypertension | | |
|  |  |  |  |  | Beta | SE | P | Beta | SE | P |
| rs1248860 | A | A | 0.516 | 0.434 | 0.010 | 0.010 | 0.772 | -0.003 | 0.001 | 0.000 |
| rs13243553 | A | A | 0.392 | 0.355 | -0.009 | 0.010 | 0.465 | -0.008 | 0.001 | 0.000 |
| rs2764261 | G | G | 0.626 | 0.578 | -0.009 | 0.010 | 0.007 | 0.027 | 0.001 | 0.000 |
| rs328902 | T | T | 0.315 | 0.302 | 0.009 | 0.011 | 0.309 | -0.011 | 0.001 | 0.000 |
| rs3781411 | T | T | 0.124 | 0.176 | -0.013 | 0.013 | 0.353 | 0.012 | 0.002 | 0.000 |
| rs6667222 | C | C | 0.252 | 0.251 | -0.009 | 0.011 | 0.189 | 0.015 | 0.002 | 0.000 |
| rs9276758 | A | A | 0.312 | 0.347 | -0.008 | 0.011 | 0.000 | 0.045 | 0.001 | 0.000 |
| SNP | effect_allele.exposure | effect_allele.outcome | eaf.exposure | eaf.outcome | Associations with vigorous physical activity | | | Associations with type 2 diabetes | | |
|  |  |  |  |  | Beta | SE | P | Beta | SE | P |
| rs1248860 | A | A | 0.516 | 0.515 | 0.010 | 0.001 | 0.000 | -0.002 | 0.008 | 0.841 |
| rs13243553 | A | A | 0.392 | 0.391 | -0.009 | 0.001 | 0.000 | 0.012 | 0.008 | 0.120 |
| rs2764261 | G | G | 0.626 | 0.627 | -0.009 | 0.001 | 0.000 | 0.012 | 0.008 | 0.152 |
| rs328902 | T | T | 0.315 | 0.315 | 0.009 | 0.001 | 0.000 | -0.009 | 0.008 | 0.264 |
| rs3781411 | T | T | 0.124 | 0.125 | -0.013 | 0.002 | 0.000 | 0.023 | 0.011 | 0.033 |
| rs6667222 | C | C | 0.252 | 0.251 | -0.009 | 0.002 | 0.000 | 0.016 | 0.009 | 0.089 |
| rs9276758 | A | A | 0.312 | 0.313 | -0.008 | 0.001 | 0.000 | 0.017 | 0.009 | 0.054 |
| SNP | effect_allele.exposure | effect_allele.outcome | eaf.exposure | eaf.outcome | Associations with vigorous physical activity | | | Associations with high cholesterol | | |
|  |  |  |  |  | Beta | SE | P | Beta | SE | P |
| rs1248860 | A | A | 0.516 | 0.603 | 0.010 | 0.001 | 0.000 | -0.236 | 0.358 | 0.509 |
| rs13243553 | A | A | 0.392 | 0.399 | -0.009 | 0.001 | 0.000 | 0.679 | 0.359 | 0.058 |
| rs2764261 | G | G | 0.626 | 0.436 | -0.009 | 0.001 | 0.000 | 0.311 | 0.374 | 0.405 |
| rs328902 | T | T | 0.315 | 0.239 | 0.009 | 0.001 | 0.000 | 0.048 | 0.403 | 0.905 |
| rs3781411 | T | T | 0.124 | 0.171 | -0.013 | 0.002 | 0.000 | 0.350 | 0.470 | 0.456 |
| rs6667222 | C | C | 0.252 | 0.332 | -0.009 | 0.002 | 0.000 | -0.188 | 0.405 | 0.643 |
| rs9276758 | A | A | 0.312 | 0.300 | -0.008 | 0.001 | 0.000 | -0.365 | 0.377 | 0.332 |
| SNP | effect_allele.exposure | effect_allele.outcome | eaf.exposure | eaf.outcome | Associations with vigorous physical activity | | | Associations with venous thromboembolism | | |
|  |  |  |  |  | Beta | SE | P | Beta | SE | P |
| rs1248860 | A | A | 0.516 | 0.514 | 0.010 | 0.001 | 0.000 | -0.001 | 0.000 | 0.054 |
| rs13243553 | A | A | 0.392 | 0.392 | -0.009 | 0.001 | 0.000 | 0.000 | 0.000 | 0.440 |
| rs2764261 | G | G | 0.626 | 0.627 | -0.009 | 0.001 | 0.000 | 0.000 | 0.000 | 0.894 |
| rs328902 | T | T | 0.315 | 0.315 | 0.009 | 0.001 | 0.000 | 0.000 | 0.000 | 0.619 |
| rs3781411 | T | T | 0.124 | 0.123 | -0.013 | 0.002 | 0.000 | 0.000 | 0.000 | 0.655 |
| rs6667222 | C | C | 0.252 | 0.252 | -0.009 | 0.002 | 0.000 | 0.000 | 0.000 | 0.432 |
| rs9276758 | A | A | 0.312 | 0.310 | -0.008 | 0.001 | 0.000 | 0.000 | 0.000 | 0.390 |
| SNP | effect_allele.exposure | effect_allele.outcome | eaf.exposure | eaf.outcome | Associations with accelerometer-based physical activity | | | Associations with coronary heart disease | | |
|  |  |  |  |  | Beta | SE | P | Beta | SE | P |
| rs11012732 | G | G | 0.332 | 0.298 | -0.225 | 0.039 | 0.000 | 0.002 | 0.011 | 0.827 |
| rs12522261 | A | A | 0.343 | 0.341 | -0.211 | 0.038 | 0.000 | 0.003 | 0.010 | 0.740 |
| rs148193266 | C | C | 0.043 | 0.043 | 0.510 | 0.092 | 0.000 | 0.033 | 0.026 | 0.203 |
| rs34517439 | A | A | 0.121 | 0.094 | -0.308 | 0.056 | 0.000 | 0.038 | 0.019 | 0.042 |
| rs56194509 | G | G | 0.220 | 0.175 | 0.303 | 0.044 | 0.000 | 0.041 | 0.014 | 0.003 |
| rs59499656 | T | T | 0.344 | 0.355 | 0.228 | 0.038 | 0.000 | 0.000 | 0.010 | 0.995 |
| rs6775319 | T | T | 0.729 | 0.684 | -0.225 | 0.041 | 0.000 | 0.005 | 0.010 | 0.594 |
| rs9293503 | C | C | 0.112 | 0.122 | -0.329 | 0.059 | 0.000 | -0.033 | 0.014 | 0.023 |
| SNP | effect_allele.exposure | effect_allele.outcome | eaf.exposure | eaf.outcome | Associations with accelerometer-based physical activity | | | Associations with ischemic heart disease | | |
|  |  |  |  |  | Beta | SE | P | Beta | SE | P |
| rs11012732 | G | G | 0.332 | 0.314 | -0.225 | 0.039 | 0.000 | 0.015 | 0.012 | 0.218 |
| rs12522261 | A | A | 0.343 | 0.320 | -0.211 | 0.038 | 0.000 | 0.001 | 0.012 | 0.922 |
| rs148193266 | C | C | 0.043 | 0.032 | 0.510 | 0.092 | 0.000 | -0.053 | 0.032 | 0.094 |
| rs34517439 | A | A | 0.121 | 0.127 | -0.308 | 0.056 | 0.000 | 0.003 | 0.017 | 0.864 |
| rs56194509 | G | G | 0.220 | 0.082 | 0.303 | 0.044 | 0.000 | 0.037 | 0.021 | 0.071 |
| rs59499656 | T | T | 0.344 | 0.418 | 0.228 | 0.038 | 0.000 | -0.012 | 0.011 | 0.268 |
| rs6775319 | T | T | 0.729 | 0.662 | -0.225 | 0.041 | 0.000 | 0.027 | 0.012 | 0.020 |
| rs9293503 | C | C | 0.112 | 0.060 | -0.329 | 0.059 | 0.000 | -0.009 | 0.024 | 0.691 |
| SNP | effect_allele.exposure | effect_allele.outcome | eaf.exposure | eaf.outcome | Associations with accelerometer-based physical activity | | | Associations with angina | | |
|  |  |  |  |  | Beta | SE | P | Beta | SE | P |
| rs11012732 | G | G | 0.332 | 0.332 | -0.225 | 0.039 | 0.000 | 0.001 | 0.000 | 0.100 |
| rs12522261 | A | A | 0.343 | 0.341 | -0.211 | 0.038 | 0.000 | 0.000 | 0.000 | 0.500 |
| rs148193266 | C | C | 0.043 | 0.043 | 0.510 | 0.092 | 0.000 | 0.001 | 0.001 | 0.370 |
| rs34517439 | A | A | 0.121 | 0.122 | -0.308 | 0.056 | 0.000 | 0.001 | 0.001 | 0.110 |
| rs56194509 | G | G | 0.220 | 0.222 | 0.303 | 0.044 | 0.000 | 0.000 | 0.000 | 0.510 |
| rs59499656 | T | T | 0.344 | 0.343 | 0.228 | 0.038 | 0.000 | -0.001 | 0.000 | 0.140 |
| rs6775319 | T | T | 0.729 | 0.731 | -0.225 | 0.041 | 0.000 | 0.001 | 0.000 | 0.005 |
| rs9293503 | C | C | 0.112 | 0.111 | -0.329 | 0.059 | 0.000 | 0.000 | 0.001 | 0.920 |
| SNP | effect_allele.exposure | effect_allele.outcome | eaf.exposure | eaf.outcome | Associations with accelerometer-based physical activity | | | Associations with Alzheimer’s disease | | |
|  |  |  |  |  | Beta | SE | P | Beta | SE | P |
| rs11012732 | G | G | 0.332 | NA | -0.225 | 0.039 | 0.000 | -0.022 | 0.017 | 0.186 |
| rs12522261 | A | A | 0.343 | NA | -0.211 | 0.038 | 0.000 | 0.023 | 0.016 | 0.163 |
| rs148193266 | C | C | 0.043 | NA | 0.510 | 0.092 | 0.000 | 0.025 | 0.042 | 0.557 |
| rs34517439 | A | A | 0.121 | NA | -0.308 | 0.056 | 0.000 | 0.046 | 0.041 | 0.260 |
| rs56194509 | G | G | 0.220 | NA | 0.303 | 0.044 | 0.000 | -0.049 | 0.020 | 0.013 |
| rs9293503 | C | C | 0.112 | NA | -0.329 | 0.059 | 0.000 | -0.074 | 0.024 | 0.002 |
| SNP | effect_allele.exposure | effect_allele.outcome | eaf.exposure | eaf.outcome | Associations with accelerometer-based physical activity | | | Associations with hypertension | | |
|  |  |  |  |  | Beta | SE | P | Beta | SE | P |
| rs11012732 | G | G | 0.332 | 0.314 | -0.225 | 0.039 | 0.000 | 0.034 | 0.011 | 0.001 |
| rs12522261 | A | A | 0.343 | 0.320 | -0.211 | 0.038 | 0.000 | 0.004 | 0.011 | 0.721 |
| rs148193266 | C | C | 0.043 | 0.032 | 0.510 | 0.092 | 0.000 | -0.030 | 0.028 | 0.278 |
| rs34517439 | A | A | 0.121 | 0.127 | -0.308 | 0.056 | 0.000 | -0.008 | 0.015 | 0.575 |
| rs56194509 | G | G | 0.220 | 0.082 | 0.303 | 0.044 | 0.000 | -0.001 | 0.018 | 0.960 |
| rs59499656 | T | T | 0.344 | 0.418 | 0.228 | 0.038 | 0.000 | -0.001 | 0.010 | 0.917 |
| rs6775319 | T | T | 0.729 | 0.662 | -0.225 | 0.041 | 0.000 | 0.033 | 0.010 | 0.001 |
| rs9293503 | C | C | 0.112 | 0.061 | -0.329 | 0.059 | 0.000 | -0.048 | 0.021 | 0.019 |
| SNP | effect_allele.exposure | effect_allele.outcome | eaf.exposure | eaf.outcome | Associations with accelerometer-based physical activity | | | Associations with type 2 diabetes | | |
|  |  |  |  |  | Beta | SE | P | Beta | SE | P |
| rs11012732 | G | G | 0.332 | 0.331 | -0.225 | 0.039 | 0.000 | -0.002 | 0.008 | 0.790 |
| rs12522261 | A | A | 0.343 | 0.341 | -0.211 | 0.038 | 0.000 | -0.017 | 0.008 | 0.039 |
| rs56194509 | G | G | 0.220 | 0.225 | 0.303 | 0.044 | 0.000 | 0.010 | 0.009 | 0.301 |
| rs59499656 | T | T | 0.344 | 0.343 | 0.228 | 0.038 | 0.000 | -0.018 | 0.008 | 0.031 |
| rs6775319 | T | T | 0.729 | 0.731 | -0.225 | 0.041 | 0.000 | 0.027 | 0.009 | 0.003 |
| rs9293503 | C | C | 0.112 | 0.106 | -0.329 | 0.059 | 0.000 | -0.024 | 0.013 | 0.059 |
| SNP | effect_allele.exposure | effect_allele.outcome | eaf.exposure | eaf.outcome | Associations with accelerometer-based physical activity | | | Associations with high cholesterol | | |
|  |  |  |  |  | Beta | SE | P | Beta | SE | P |
| rs11012732 | G | G | 0.332 | 0.255 | -0.225 | 0.039 | 0.000 | -0.246 | 0.410 | 0.549 |
| rs12522261 | A | A | 0.343 | 0.303 | -0.211 | 0.038 | 0.000 | -0.035 | 0.376 | 0.925 |
| rs148193266 | C | C | 0.043 | 0.012 | 0.510 | 0.092 | 0.000 | 0.241 | 1.673 | 0.885 |
| rs34517439 | A | A | 0.121 | 0.037 | -0.308 | 0.056 | 0.000 | 1.387 | 0.932 | 0.137 |
| rs56194509 | G | G | 0.220 | 0.105 | 0.303 | 0.044 | 0.000 | -0.011 | 0.571 | 0.984 |
| rs9293503 | C | C | 0.112 | 0.067 | -0.329 | 0.059 | 0.000 | 0.204 | 0.720 | 0.777 |
| SNP | effect_allele.exposure | effect_allele.outcome | eaf.exposure | eaf.outcome | Associations with accelerometer-based physical activity | | | Associations with venous thromboembolism | | |
|  |  |  |  |  | Beta | SE | P | Beta | SE | P |
| rs11012732 | G | G | 0.332 | 0.331 | -0.225 | 0.039 | 0.000 | 0.001 | 0.000 | 0.001 |
| rs12522261 | A | A | 0.343 | 0.343 | -0.211 | 0.038 | 0.000 | 0.000 | 0.000 | 0.788 |
| rs148193266 | C | C | 0.043 | 0.044 | 0.510 | 0.092 | 0.000 | -0.001 | 0.001 | 0.037 |
| rs34517439 | A | A | 0.121 | 0.125 | -0.308 | 0.056 | 0.000 | 0.001 | 0.000 | 0.170 |
| rs56194509 | G | G | 0.220 | 0.222 | 0.303 | 0.044 | 0.000 | 0.001 | 0.000 | 0.014 |
| rs59499656 | T | T | 0.344 | 0.344 | 0.228 | 0.038 | 0.000 | -0.001 | 0.000 | 0.030 |
| rs6775319 | T | T | 0.729 | 0.733 | -0.225 | 0.041 | 0.000 | 0.000 | 0.000 | 0.203 |
| rs9293503 | C | C | 0.112 | 0.109 | -0.329 | 0.059 | 0.000 | 0.000 | 0.000 | 0.955 |

SE, Standard error; SNP, Single-nucleotide polymorphism.
