# Supplementary material for: Physical activity and the risk of developing 8 age-related diseases: epidemiological and Mendelian randomization studies
Source: Eur Rev Aging Phys Act. 2024 Sep 18;21:24. doi: 10.1186/s11556-024-00359-2 (PMC11412029; doi:10.1186/s11556-024-00359-2)
Supplement: Supplementary file 8 — Supplementary Material 8. [file 11556_2024_359_MOESM8_ESM.docx]

Supplemental Table 2 Details of studies and datasets used for analyses

| Exposure/Outcomes | ID in MR base | Pubmed ID | First author | Consortium | Sample size | Year | Sex | Participants |
| --- | --- | --- | --- | --- | --- | --- | --- | --- |
| Accelerometer-based physical activity | ebi-a-GCST006099 | 29899525 | Klimentidis YC | NA | 91084 | 2018 | NA | European |
| Vigorous physical activity | ebi-a-GCST006098 | 29899525 | Klimentidis YC | NA | 261055 | 2018 | NA | European |
| Coronary heart disease | ieu-a-7 | 26343387 | Nikpay | CARDIoGRAMplusC4D | 184305 | 2015 | Males and Females | Mixed |
| Ischemic heart disease | finn-b-I9_ISCHHEART | NA | NA | NA | 218792 | 2021 | Males and Females | European |
| Angina | ukb-b-8468 | NA | Ben Elsworth | MRC-IEU | 461880 | 2018 | Males and Females | European |
| Alzheimer’s disease | ieu-a-297 | 24162737 | Lambert | IGAP | 54162 | 2013 | Males and Females | European |
| Hypertension | finn-b-I9_HYPTENSESS | NA | NA | NA | 205694 | 2021 | Males and Females | European |
| Type 2 diabetes | ebi-a-GCST006867 | 30054458 | Xue A | NA | 655666 | 2018 | NA | European |
| High cholesterol (LDL cholesterol) | ebi-a-GCST008045 | NA | Howe LJ | Within family GWAS consortium | 29212 | 2022 | Males and Females | European |
| Venous thromboembolism | ukb-d-I9_VTE | NA | Neale lab | NA | 361194 | 2018 | Males and Females | European |

NA, Not available.
