# Supplementary material for: Physical activity and the risk of developing 8 age-related diseases: epidemiological and Mendelian randomization studies
Source: Eur Rev Aging Phys Act. 2024 Sep 18;21:24. doi: 10.1186/s11556-024-00359-2 (PMC11412029; doi:10.1186/s11556-024-00359-2)
Supplement: Supplementary file 9 — Supplementary Material 9. [file 11556_2024_359_MOESM9_ESM.doc]

Supplemental Table 3 A glossary of terms involved in this study

| Term | Explanation |
| --- | --- |
| Inverse causality | Reverse causality is a form of confounding that is difficult to account for. It arises if the outcome or preclinical aspects of the disease that lead to the outcome affect the risk factor. People with symptoms of cardiovascular disease, for example, may consume less alcohol than those without symptoms. This would lead to a negative association between a risk factor (alcohol) and an outcome (cardiovascular disease). Interpreting this as being because alcohol consumption decreases the risk of cardiovascular disease would be misleading. |
| Mendelian randomization (MR) | A method that uses genetic variation to strengthen possible causal inference regarding modifiable exposures influencing risk of disease or other outcomes. Most MR studies are implemented within an instrumental variable framework, using genetic variants as instrumental variables. |
| Instrumental variables (IVs) | Variables associated with the exposure of interest, that are not related to confounders, and that affect the outcome only through the exposure. |
| Genetic variant | A variation in the DNA sequence that is found within a population. Typically, a single-nucleotide polymorphism. |
| Single-nucleotide polymorphism (SNP) | A genetic variant in which a single base pair in the DNA varies across the population, at an appreciable frequency. SNPs typically have 2 alleles (eg, adenine, cytosine, guanine, or will be associated with a higher value of the trait, the other with a lower value. In MR studies, SNPs are the most common thiamine). If the SNP is associated with the trait, then 1 allele genetic variants used as IVs for a modifiable exposure. |
| Horizontal pleiotropy | A situation in which genetic variants affect the outcome via pathways independent of the exposure. This is a violation of the exclusion restriction assumption and a source of bias in MR studies. |
| Exercise unit (EU) | Total physical activity score was assessed with the CARDIA Physical Activity History Questionnaire, an interviewer-administered self-report of frequency of participation in each of 13 categories of sports and exercise during the previous 12 months. The 13 categories included 8 vigorous-intensity activities (running or jogging; racquet sports; biking; swimming; exercise or dance class; job lifting, carrying, or digging; shoveling or lifting during leisure; and strenuous sports) and 5 moderate-intensity activities (nonstrenuous sports, walking and hiking, golfing and bowling, home exercises or calisthenics, and home maintenance or gardening). The score are expressed in 'exercise units' (EU). For reference, a total activity score of 300 exercise units approximates 150 minutes of moderate-intensity activity per week. |
